# Balanced below- and above-ground growth improved yield and water productivity by cultivar renewal for winter wheat

**DOI:** 10.3389/fpls.2022.1022023

**Published:** 2022-10-31

**Authors:** Haotian Li, Lu Li, Na Liu, Zimeng Liu, Yang Lu, Liwei Shao

**Affiliations:** ^1^ Key Laboratory of Agricultural Water Resources, The Center for Agricultural Resources Research, Institute of Genetics and Developmental Biology, The Chinese Academy of Sciences, Shijiazhuang, China; ^2^ University of Chinese Academy of Sciences, Beijing, China

**Keywords:** water deficit, yield components, biomass accumulation_3_, root: shoot ratio, root efficiency

## Abstract

Breeding cultivars that can maintain high production and water productivity (WP) under various growing conditions would be important for mitigating freshwater shortage problems. Experiments were carried out to assess the changes in yield and WP of different cultivars by breeding and traits related to the changes using tubes with 1.05 m depth and 19.2 cm inner diameter buried in the field located in the North China Plain. Six winter wheat cultivars released from the 1970s to 2010s were assessed under three water levels for three seasons. The results indicated that yield was on average improved by 19.9% and WP by 21.5% under the three water levels for the three seasons for the cultivar released in the 2010s as compared with that released in the 1970s. The performance of the six cultivars was relatively stable across the experimental duration. The improvement in yield was mainly attributed to the maintenance of higher photosynthetic capacity during the reproductive growth stage and greater above-ground biomass accumulation. These improvements were larger under wet conditions than that under dry conditions, indicating that the yield potential was increased by cultivar renewal. Traits related to yield and WP improvements included the increased harvest index and reduced root: shoot ratio. New cultivars reduced the redundancy in root proliferation in the topsoil layer, which did not compromise the efficient utilization of soil moisture but reduced the metabolic input in root growth. Balanced above- and below-ground growth resulted in a significant improvement in root efficiency at grain yield level up to 40% from the cultivars released in the 1970s to those recently released. The results from this study indicated that the improved efficiency in both the above- and below-parts played important roles in enhancing crop production and resource use efficiency.

## 1 Introduction

Aggravated water deficit is one of the limiting factors of wheat growth and productivity in many regions, including the North China Plain (NCP) (Alia et al., 2019; Motazedian et al., 2019; Yao et al., 2019). Due to the shortage of precipitation during the winter wheat growing season, supplementary irrigation is necessary to obtain high and stable yield in the NCP (Fan et al., 2019). The impacts of water stress on grain yield depended on complex growth processes of below- and above-ground parts of crops, influenced by the intensity of soil water deficit and genetic background of wheat cultivars (Nguyen et al., 2022). In response to water deficit, the plants will continue to exhaust its water residuals through root proliferation as phenotypic stability (Nicotra et al., 2010). After the soil moisture was depleted, stomatal conductance of leaves thereby decreasing transpiration and photosynthesis rate, shrinking the relative growth rate of crops and ultimately limiting dry matter accumulation as plasticity responses (Sade et al., 2018; Bacher et al., 2021).

Breeding cultivars with enhanced drought tolerance performance in key growth periods through natural and artificial selection was critical for improving yield potential and water productivity of crops (Senapati et al., 2019). Identifying the various mechanisms that adapt to the yield reduction caused by drought stress among different wheat cultivars is important to improve water productivity during breeding processes (Bailey-Serres et al., 2019; Zeng et al., 2021). Under water-limited conditions, achieving high biomass and harvest index (HI) values are crucial for yield improvement (Zhang et al., 2010; Deery et al., 2016; Thapa et al., 2019). Changes in dry matter allocation between below- and above-ground affected crop production among different wheat cultivars (Zhu et al., 2022). The influence of the environment on morpho-physiological traits is relatively low and predictable, resulting in higher genetic progress compared to yield-based selection (Fischer et al., 2012). Thus, indirect selection of traits related to drought tolerance and high resource use efficiency during the breeding process is an analytic approach that involves understanding interrelationships among various attributes and responses to environmental variation (Li et al., 2018; Pouri et al., 2019; Rezzouk et al., 2022). Meta-analysis has indicated that the breeding process of high-yield dryland winter wheat has been continuously enhancing drought tolerance (high leaf water potential and osmotic adjustment, with a small root system) while weakening drought avoidance (large root biomass, small leaf area, and reduced stomatal conductance under water deficits) of plants (Li et al., 2021). For above-ground parts of crops, flag leaf is the main component of the canopy at the reproductive stage of winter wheat, and it is an important organ to determine the grain- filling rate and final yield (Vicentea et al., 2018). Physiological indicators such as net photosynthetic rate and stomatal conductance have been used to clarify the water demand and dry matter allocation of crops at different stages at the leaf level (Ruszkowski et al., 2015; Costa et al., 2019; Lamptey et al., 2020).

Traits constituting yield were interrelated with each other and influenced by morpho-physiological traits such as phenology parameters, plant structure and physiological activity among different wheat cultivars (El Mohsen et al., 2012). The current breeding processes mainly aimed at specific physiological and agronomic characteristics contributes to the improvement of yield and drought tolerance of wheat cultivars, while yield performance under water stress conditions was determined by coordination mechanisms between below- and the above-ground parts of the plant (Xie et al., 2017; Figueroa-Bustos et al., 2020; Ober et al., 2021). Both natural and artificial selection of wheat cultivars with high yield and drought resistance processes numerous physiological and molecular mechanisms adapt to water stress while realizing the potential yield (Liu and Qin, 2021).

The hypothesis of this study was that the breeding processes for winter wheat from the 1970s to 2010s increased the yield potential of wheat cultivars by optimizing both below- and above-ground growth. Improvement in grain yield and WP by cultivar renewal depends on balancing the growth of above-ground and below-ground parts of plants, characterized by higher root system efficiency and harvest index to achieve high yield without increasing carbon cost.

## 2 Materials and methods

### 2.1 Experimental design

Tube experiments were conducted at the Luancheng Agro-Eco-Experimental station of the Chinese Academy of Sciences (37^°C^53′ N, 114^°C^40′ E; 50 m asl) located in the northern part of the NCP during 2017-2020 for three winter wheat growing seasons. The PVC tubes (1.05 m length with a 19.2 cm inner diameter) were buried flush with the soil surface surrounded by field-grown winter wheat to keep the growing conditions of the crop and the soil temperature in the tubes consistent with that in the surrounding field. There was a movable shed used during rainfall events to prevent rain, therefore all the water supply came from irrigation. The tubes were filled with soil obtained from the tillage layer of 0-20 cm in the surrounding field. The soil properties and nutrient contents are shown in **Table 1**. The initial soil water content for all tubes was set at 0.21 g g^-1^ (87.5% of field capacity). Soil was packed into tubes with a bulk density of 1.4 g cm^-3,^ and the bottom of the tubes was sealed with plastic film. The total soil depth for each tube was 1 m, and there was 5 cm depth without soil for the top of the tubes for water supply.

**Table 1:** Soil properties and nutrient contents for the soils collected from the field and used to fill the tubes.

SoilTexture	Field capacity(g/g)	Wilting point(g/g)	Organic matter(g/kg)	Total N(g/kg)	Ava. N(mg/kg)	Ava. P(mg/kg)	Ava. K(mg/kg)
Loam	0.24	0.09	18.9	1.1	89.7	20.5	95.6

The top 10 cm soil layer of the tubes was mixed with 5.0 g of diammonium (containing 46% P_2_O_5_ and 16% N), 2.0 g of urea (containing 46% N) and 6.0 g potassium chloride (containing 60% K_2_O) as base fertilizers before filling. The cultivars used in this study were released in different years and had been widely cultivated locally, i.e., *Jimai 7* released in 1976, *Jiami 26* in 1988, *Jiami 30* in earlier 1992, *Shi 4185* in late 1999, *Kenong 199* in 2006 and *Shimai 22* in 2013, reflecting the general trend of the drought-resistant and high-yield breeding processes of winter wheat cultivars in Hebei province in the NCP. Thirty seeds were selected and planted into each tube manually on approximately the 15^th^ of October. After sowing, the soil surface was covered with 1 cm fine sand to reduce soil evaporation. At the three-leaf stage, seedlings were thinned to 20 per tube.

The seasonal irrigation amount was set at 320 mm for the high-level water supply (HW), 240 mm for the medium-level water supply (MW) and 180 mm for the lower-level water supply (LW). There were 18 treatments for six winter wheat cultivars under the three irrigation treatments. Each treatment had four replicates, and 72 tubes were used in total. Tubes were randomly arranged in the field.

The irrigation schedule for the three irrigation treatments is listed in **Table 2**. The total irrigation amount was equally divided into several irrigations, applied at key growing stages including jointing (approximately 160 days after sowing, DAS), heading (approximately 190 DAS), anthesis (approximately 200 DAS) and grain filling (approximately 208 DAS). At the jointing stage, 2.0 g urea was applied to each tube by dissolving into the irrigation water. All tubes were harvested on approximately June 10^th^.

**Table 2:** Irrigation schedules for the three water treatments.

Irrigation treatments	Irrigation timing and amount (mm)				Total irrigation (mm)
	Jointing	Heading	Anthesis	Grain filling	
High-level water supply (HW)	80	80	80	80	320
Medium-level water supply(MW)	80	0	80	80	240
Low-level water supply (LW)	80	0	80	0	160

### 2.2 Measurements

#### 2.2.1 Phenology and leaf photosynthesis

During the three growing seasons of winter wheat, the phenology parameters of all tubes were regularly monitored. Pests, diseases, and weeds were prevented by spraying the same pesticides as the surrounding fields to ensure the crops being not affected by those factors. During the heading (around 25^th^ of April) and grain filling stages (around 15^th^ of May), four fully expanded flag leaves of each tube were randomly selected to measure the leaf photosynthesis rate (Pn) by a CO_2_ gas exchange system (LI-6400, LI-COR Inc., USA) from 10:00 to 14:00 h local time on sunny days. The four leaves were randomly selected from each replicate of the treatments.

#### 2.2.2 Biomass, grain yield and yield components

At harvest, plants in each tube were manually cut from the ground, plant height and the spike numbers of each tube were measured. After air-drying to a constant weight (80°C for 48 hours), the total biomass of above-ground part of plant was weighed. A thresher was used to separate grains from straw, the weight of the grains (at 13% of water contents) was obtained, and seed numbers were counted by an automatic grain counting machine. The mean environmental yield was calculated as the seasonal average grain yield of the six wheat cultivars under each irrigation treatment. The harvest index (HI) was calculated as grain yield/total above-ground biomass.

#### 2.2.3 Soil water contents and root measurements at harvest

After the above-ground parts of the plants were harvested, the PVC tubes were excavated and separated into 20 cm thick segments from top to bottom. Approximately 100 g of soil sample without roots was removed from each segment for soil water content measurement by the oven-drying method. The rest of each soil column was washed to separate roots, and roots were oven dried at 80 °C for 24 hours to determine dry weight. The root weight density (RWD) was calculated as root weight/soil volume, and the root: shoot ratio (RS) was calculated as total root weight/above-ground biomass. Root system efficiency (RSE) in this study was defined as grain yield per unit root weight.

#### 2.2.4 Evapotranspiration and water productivity

Seasonal evapotranspiration (ET) was calculated using the following equation:


(1) ET=P+I+SWD+CR-R-D

where ET is the total seasonal evapotranspiration; P is precipitation (mm); I is the irrigation water (mm); SWD (mm) is the soil water depletion, which is defined as the soil water content (SWC) of the whole column at sowing after subtracting that at harvesting; CR is the amount of water rising from the soil capillary to the root zone (mm); R is surface runoff (mm); and D is the drainage of the soil water from the root zone (mm). P, D, R and CR were taken as zero under the experimental conditions of this study. Water productivity (WP) at the grain level was calculated as grain yield/seasonal ET.

### 2.3 Data analysis

Microsoft Excel 2021 (Microsoft, Redmond, USA) and IBM SPSS statistics 26 (IBM, Stanford, USA) were used for data analysis. Correlations among different parameters were analyzed based on the average values of three growing seasons for each treatment. The standard deviation of each parameter among different treatments was obtained from the average values of the three growing seasons. When the variance homogeneity test was successful, the least significant difference (LSD) test was performed (P < 0.05).

## 3 Results

### 3.1 Grain yield and water productivity among the six cultivars

**Figure 1:**
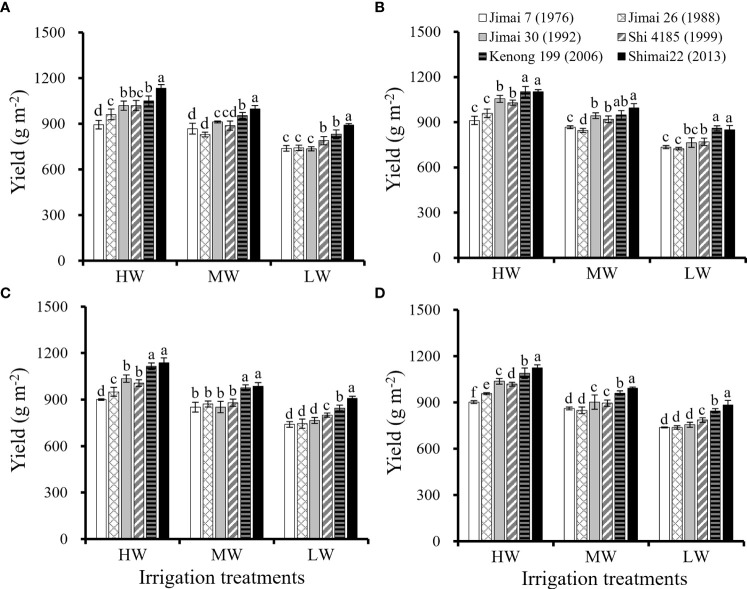
Grain yield for the six winter wheat cultivars released in different years for each of the growing season (**A–C**, respectively) and the average values for the three seasons **(D)** under the three irrigation treatments (Means ‘±’ standard deviation with different letters for the same irrigation treatment differ significantly at P < 0.05. HW, high-level water supply; MW, medium-level water supply; and LW, low-level water supply).

The yields of the six wheat cultivars under different irrigation treatments in the three seasons are shown in **Figure 1**. With the renewal of winter wheat cultivars, the yield of new cultivars in the same growing season was significantly higher than that of the old cultivars, and the improvement in yield was most significant under the condition of HW. The yield of wheat cultivars decreased significantly with the reduction in irrigation amount. The average values of environmental yield were 1021.4 g m^-2^ under HW, 910.3 g m^-2^ under MW and 790.9 g m^-2^ under LW. Compared with the old cultivar ‘Jimai 7’, the yield of new cultivar’Shimai 22’ increased by 24.6% under HW, 15.2% under MW and 19.8% under LW, indicating that the yield potential was improved by cultivar renewing especially under relatively sufficient water supply.

**Figure 2:**
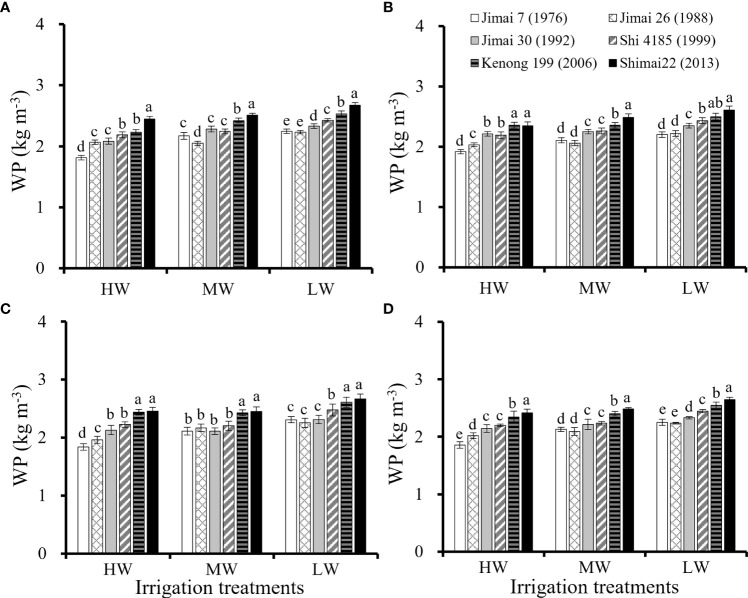
Water productivity (WP) in each of the three growing seasons (**A–C**, respectively) and the average values for the three seasons **(D)** for six winter wheat cultivars released in different years under the three irrigation treatments (Means ‘±’ standard deviation with different letters for the same irrigation treatment differ significantly at P < 0.05. HW, high-level water supply; MW, medium-level water supply; and LW, low-level water supply).

The SWD and ET of wheat cultivars (**Table 3**) was affected by the water supply condition. The average seasonal ET for the six cultivars ranged from 462.7 to 486.0 mm under HW, from 399.7 to 407.2 mm under MW, and from 321.3 to 336.5 mm under LW. Similar water consumption was found among the six cultivars under the same water level, but with a large variation in grain yield, resulting in a large variation in WP for the six cultivars (**Figure 2**). The environmental average WP in the three seasons was 2.16 kg m^-3^ under HW, 2.26 kg m^-3^ under MW and 2.41 kg m^-3^ under LW. There was a slight increase in WP from HW to LW, indicating that water stress increased the overall WP. Compared with the old cultivar ‘Jimai 7’, the WP of the new cultivar ‘Shimai 22’ increased by 30.1% under HW, 16.6% under MD and 17.8% under LD. WP was significantly improved with renewal in cultivars, similar to grain production.

**Table 3:** The average values of soil water depletion (SWD) and seasonal evapotranspiration (ET) of the six winter wheat cultivars released in different years under the three irrigation treatments for the three growing seasons *.

Cultivars	High-level water supply		Medium-level water supply		Low-level water supply	
	SWD (mm)	ET (mm)	SWD (mm)	ET (mm)	SWD (mm)	ET (mm)
Jimai 7	166.0 ± 9.3^a^	486.0 ± 9.3	164.6 ± 6.1^a^	404.6 ± 6.1	167.6 ± 6.5^ab^	327.6 ± 6.5
Jimai 26	157.9 ± 5.9^a^	477.9 ± 5.9	165.8 ± 4.2^a^	405.8 ± 4.2	169.7 ± 3.2^ab^	329.7 ± 3.2
Jimai 30	164.0 ± 6.8^a^	484.0 ± 6.8	167.2 ± 10.3^a^	407.2 ± 10.3	164.0 ± 7.8^ab^	324.0 ± 7.8
Shi 4185	142.7 ± 9.6^b^	462.7 ± 9.6	160.3 ± 5.3^a^	400.3 ± 5.3	161.3 ± 4.6^b^	321.3 ± 4.6
Kenong 199	145.1 ± 7.0^b^	465.1 ± 7.0	159.7 ± 4.8^a^	399.7 ± 4.8	172.0 ± 10.4^a^	332.0 ± 10.4
Shimai 22	145.2 ± 4.0^b^	465.2 ± 4.0	159.9 ± 2.0^a^	399.9 ± 2.0	176.5 ± 3.6^a^	336.5 ± 3.6

*: ‘±’ represents the standard deviation obtained from the average values of the three growing seasons, different lowercase letter indicates the significant difference among wheat cultivars at same irrigation level at P < 0.05.

**Figure 3:**
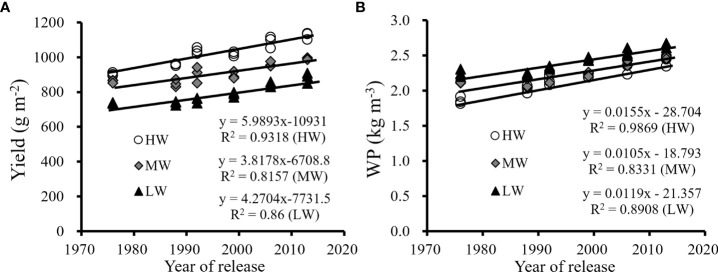
The changes in the average yield **(A)** and WP **(B)** of three seasons with the year of release of six winter wheat cultivars under three water supply levels (HW, high-level water supply; MW, medium-level water supply; and LW, low-level water supply).

The trends in yield and WP improvement with renewal in cultivars were similar, as shown in **Figure 3**. Continuous improvements in yield and WP were obtained with the cultivars released from the 1970s to 2010s. The results also indicated that the improvements in yield and WP were affected by the water levels. The average improvements in grain yield by cultivar renewal were 5.99 g/m^2^/a under HW, 3.82 g/m^2^/a under MW and 4.27 g/m^2^/a under LW. The average improvements for WP were 0.012 kg/m^3^/a under HW, 0.011 kg/m^3^/a under MW, and 0.016 kg/m^3^/a under LW. The results showed that yield improvements were greater under HW than that under the MW and LW treatments by cultivar renewal, while the improvement in WP was greater under LW than under HW and MW. New cultivars had better environmental adaptability to achieve their yield potentials under various water supply conditions and have the advantage of efficiently utilizing water under drier conditions characterized by higher WP.

### 3.2 Changes in yield components and above-ground biomass production

**Table 4** shows the three yield components (spikes per area, seeds per spike and 1000-seed weight) for the six wheat cultivars under different water treatments during the three seasons. The yield reduction from HW to MW and from HW to LW could attribute to the reduction in spikes per area (from 6.1-7.8%), seeds per spike (2.1-9.8%) and 1000-seed weight (2.7-6.7%). The yield increase from cultivar renewal could be attributed to the increase in 1000-seed weight under various water supply conditions (R^2^ = 0.53-0.87, significant at P < 0.05 and P < 0.01), followed by the seeds per spike under HW supply (R^2^ = 0.56; significant at P < 0.05). The 1000-seed weight of ‘Shimai 22’ was 7.9%-10.3% higher than the environmental average value under the different water treatments.

**Table 4:** The average values of the three yield components for the six winter wheat cultivars released in different years under the three irrigation treatments for the three growing seasons *.

Cultivars	High-level water supply				Medium-level water supply				Low-level water supply			
	Spikes per area (m^2^)	Seeds per spike	1000-seed weight (g)	Spikes per area (m^2^)		Seeds per spike	1000-seed weight (g)	Spikes per area (m^2^)		Seeds per spike	1000-seed weight (g)
Jimai 7	987.7 ± 18.0^ab^	23.2 ± 0.1^b^	39.4 ± 0.9^b^	967.6 ± 25.9^b^		23.8 ± 0.6^c^	37.5 ± 0.5^b^	944.5 ± 20.0^a^		22.4 ± 0.5^b^	34.8 ± 0.1^c^
Jimai 26	996.4 ± 27.8^ab^	25.9 ± 0.9^a^	37.1 ± 0.9^c^	871.6 ± 24.2^c^		26.1 ± 0.7^ab^	37.3 ± 0.6^b^	921.5 ± 13.2^a^		23.4 ± 0.2^a^	34.2 ± 0.6^c^
Jimai 30	1013.7 ± 20.0^a^	26.6 ± 0.7^a^	38.5 ± 0.8^b^	953.2 ± 18.0^b^		25.1 ± 0.2^b^	37.8 ± 0.4^b^	915.7 ± 15.4^a^		23.5 ± 0.4^a^	35.0 ± 1.0^bc^
Shi 4185	1007.9 ± 13.2^a^	26.0 ± 0.6^a^	38.9 ± 0.3^b^	1019.1 ± 1.8^a^		25.2 ± 0.6^b^	34.9 ± 0.7^c^	935.9 ± 5.0^a^		23.0 ± 0.5^ab^	36.6 ± 0.4^b^
Kenong 199	930.1 ± 10.0^c^	26.8 ± 0.4^a^	43.7 ± 0.9^a^	826.5 ± 13.2^d^		26.5 ± 0.5^a^	43.8 ± 0.8^a^	840.9 ± 13.2^c^		23.5 ± 0.2^a^	42.8 ± 0.3^a^
Shimai 22	970.5 ± 18.0^b^	26.8 ± 0.2^a^	43.3 ± 0.3^a^	910.0 ± 14.1^c^		25.4 ± 0.1^b^	43.0 ± 0.1^a^	886.9 ± 3.6^b^		24.1 ± 0.8^a^	41.3 ± 1.2^a^

*: ‘±’ represents the standard deviation obtained from the average values of the three growing seasons, different lowercase letter indicates the significant difference among wheat cultivars at same irrigation level at P < 0.05.

The seed weight of new cultivars was less affected by water supply. For example, the 1000-seed weight of ‘Shimai 22’ decreased by 0.66% and 4.58% under MW and LW, respectively, compared with that under HW, while ‘Jimai 7’ decreased by 4.86% and 11.63%, respectively. The results indicated that the grain weight of the new cultivars was higher and stable than that of the old cultivars.

**Figure 4:**
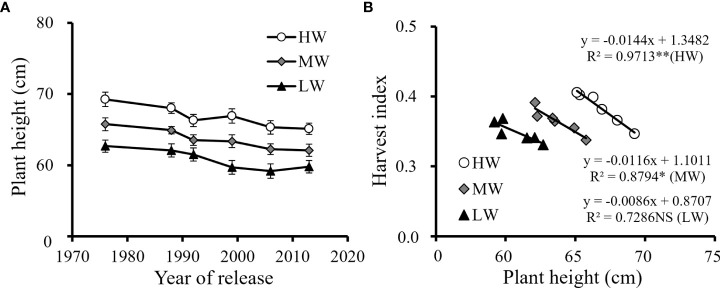
Plant height changes with the year of release of six cultivars **(A)** and the correlation of plant height with the harvest index **(B)** under three water supply conditions averagely for the three growing seasons (NS, no significant relationship; **, significant at P < 0.01 and *, significant at P < 0.05. HW, high-level water supply; MW, medium-level water supply; and LW, low-level water supply).

Plant height showed a declining trend from the old cultivars to the recent cultivars (**Figure 4A**). Breeding processes reduced the plant height of the new cultivars. With the reduction in plant height, the HI increased by 11.4-17.0% from the old to the new ones, and negative correlations were found between plant height and HI under the three water levels (**Figure 4B**). The results indicated that the cultivar renewing characterized by decreased plant height allowed more biomass allocated to seeds, which was manifested as the improvement in grain yield and HI.

**Figure 5:**
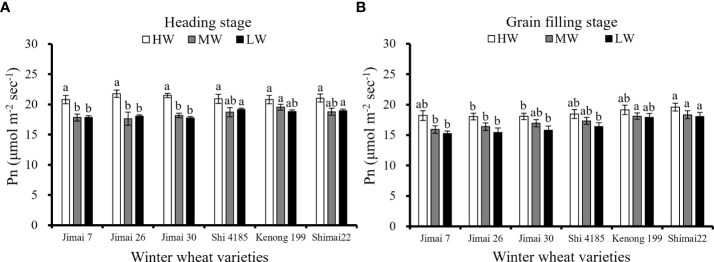
Average Pn at heading **(A)** and grain filling stage **(B)** under different irrigation treatments for the three growing seasons (Means ‘±’ standard deviation with different letters for the same irrigation treatment differ significantly at P < 0.05. HW, high-level water supply; MW, medium-level water supply; and LW, low-level water supply).

There was no significant difference in the Pn of flag leaves among the six wheat cultivars at the heading stage under HW (**Figure 5A**), but a significant difference was found under MW, LW and all water supply levels during the later grain filling stage (**Figure 5B**). The Pn of flag leaves were significantly affected by the decreased of water supply level. Since both MW and LW were not irrigated at heading stage, there were no significant difference between mean environmental Pn of two treatments. The Pn of the new cultivar ‘Shimai 22’ was significantly greater than that of the old cultivar ‘Jimai 7’ during the grain filling stage under the three water levels. Compared with the Pn at the grain filling stage under HW, the reduction in the flag leaf Pn under LW was 16.4% for ‘Jimai 7’, 14.6% for ‘Jimai 26’, 12.5% for ‘Jimai 30’, 11.2% for ‘Shi 4185’, 6.6% for ‘Kenong 199’ and 8.0% for ‘Shimai 22’. The results indicated that the sensitivity of flag leaf Pn to water stress was lower in new wheat cultivars than that in the earlier-released wheat cultivars.

**Figure 6:**
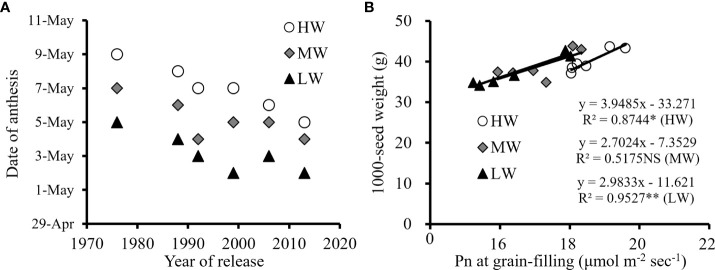
Average date of anthesis among wheat cultivars with year of release **(A)** and correlation of average Pn at grain filling growing stage with 1000-seed weight **(B)** under different irrigation treatments for the three growing seasons (NS, no significant relationship; **, significant at P < 0.01 and *, significant at P < 0.05. HW, high-level water supply; MW, medium-level water supply; and LW, low-level water supply).

Under MW and LW, the Pn at the heading growing stage of new winter wheat cultivars was relatively higher than that of old cultivars. The higher metabolism of new cultivars during vegetative growth accelerated the growth of plants characterized by earlier anthesis (**Figure 6A**); thus, prolonging the grain filling period combined with higher leaf Pn contributed to dry matter accumulation. The biomass production increased by 6.6-12.0% from the old to the new cultivars under the three water levels, and higher biomass allocation during the reproductive stage was important for the 1000-seed weight and yield improvement of new wheat cultivars (**Figure 6B**). Under the LW, the Pn at the grain filling growing stage of the new cultivar ‘Shimai 22’ was 18.3% higher than that of the old cultivar ‘Jimai 7’, while the improvement in the corresponding 1000-seed weight was 18.6% (**Table 4**).

**Figure 7:**
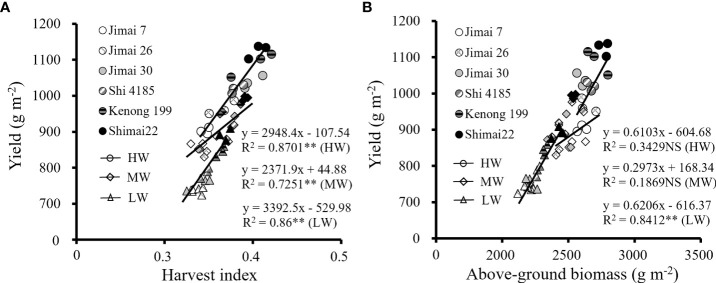
Correlation of average value of harvest index **(A)** and above-ground biomass **(B)** among each wheat cultivar with grain yield for the three growing seasons (NS, no significant relationship; **, significant at P < 0.01 and *, significant at P < 0.05. HW, high-level water supply; MW, medium-level water supply; and LW, low-level water supply).

Water supply levels had significant effects on above-ground biomass accumulation (AGB) and HI among wheat cultivars (**Table 5**). Both HI and AGB were effective factors to evaluate the ability of biomass accumulation and allocation to grains, and positive correlations existed between the HI (**Figure 7A**) and AGB (**Figure 7B**) with grain yield. Under HW, the yield change from the old cultivars to new ones was in the range of -6.9% to 12.6% as compared to the environmental background average yield, while the variation range of HI was from -5.1% to 5.8%, and the corresponding AGB changed from -1.8% to 7.0%. The results showed that the yield improvement with cultivars renewing of winter wheat depended on higher HI and AGB under water stress conditions.

**Table 5:** The average values of above-ground biomass (AGB) and harvest index (HI) for the six winter wheat cultivars released in different years under the three irrigation treatments for the three growing seasons *.

Cultivars	High-level water supply		Medium-level water supply		Low-level water supply	
	AGB (g m^-2^)	HI	AGB (g m^-2^)	HI	AGB (g m^-2^)	HI
Jimai 7	2602.9 ± 35.8^bc^	0.35 ± 0.01^c^	2553.1 ± 66.6^ab^	0.34 ± 0.01^c^	2228.7 ± 9.4^d^	22.4 ± 0.00^c^
Jimai 26	2640.9 ± 59.6^bc^	0.37 ± 0.01^bc^	2393.4 ± 45.3^b^	0.35 ± 0.01^bc^	2159.7 ± 38.7^e^	23.4 ± 0.00^b^
Jimai 30	2600.7 ± 31.7^c^	0.40 ± 0.01^ab^	2476.5 ± 45.5^b^	0.36 ± 0.02^bc^	2217.6 ± 42.8^cd^	23.5 ± 0.01^b^
Shi 4185	2671.5 ± 27.6^b^	0.38 ± 0.01^b^	2426.0 ± 48.7^b^	0.37 ± 0.01^b^	2269.2 ± 12.6^c^	23.0 ± 0.00^b^
Kenong 199	2713.8 ± 77.3^ab^	0.40 ± 0.02^ab^	2585.4 ± 50.0^a^	0.37 ± 0.01^ab^	2324.2 ± 15.5^c^	23.5 ± 0.00^a^
Shimai 22	2771.5 ± 36.2^a^	0.41 ± 0.01^a^	2537.8 ± 15.1^ab^	0.39 ± 0.00^a^	2419.4 ± 44.6^a^	24.1 ± 0.01^a^

*: ‘±’ represents the standard deviation obtained from the average values of the three growing seasons, different lowercase letter indicates the significant difference among wheat cultivars at same irrigation level at P < 0.05.

### 3.3 Changes in below-ground growth related to cultivar renewal

**Figure 8:**
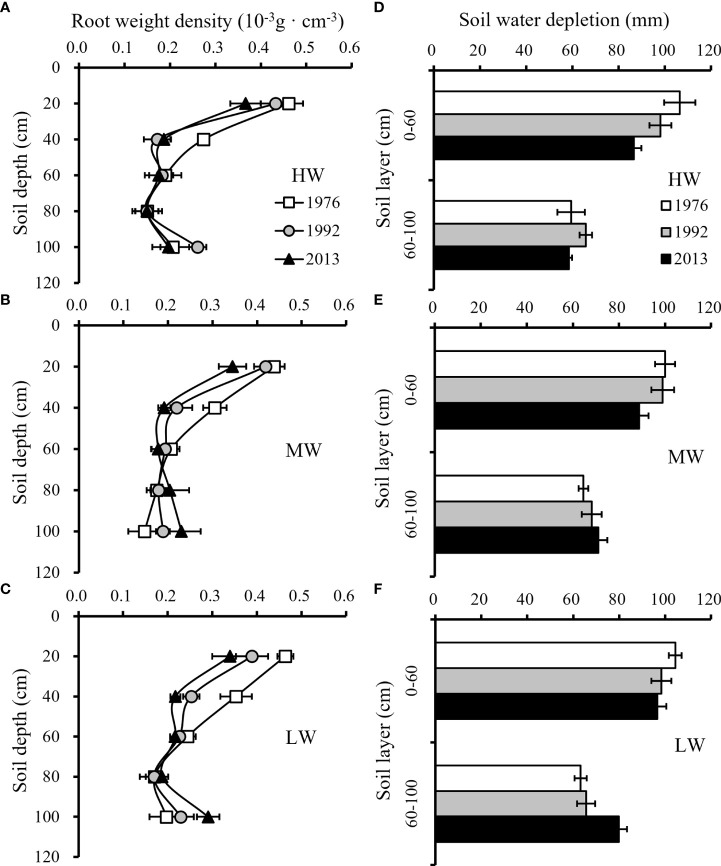
Distribution of average root weight density (RWD) along soil profiles **(A-C)** and average soil water depletion (SWD) in different soil layers **(D-F)** among three wheat cultivars released in different years under three irrigation treatments for the three growing seasons (HW, high-level water supply; MW, medium-level water supply; and LW, low-level water supply).

Three wheat cultivars, ‘Jimai 7’, ‘Jimai 30’ and ‘Shimai 22’, with release year of 1976, 1992 and 2013 respectively were selected to analyze the root distribution along the soil profiles (**Figures 8A–C**) and the SWD at different soil layers (**Figures 8D–F**). Water stress generally promotes the growth of the root system to take up more water to meet crop water demand. The average total root weight among wheat cultivars increased from 231.9 g/m^2^ to 260.9 g/m^2^ from the HW to LW treatment, while the average soil water depletion during the growing season was 153.5 mm for HW, 162.9 mm for MW and 168.5 mm for LW, corresponding to 91.4%, 97% and 100% depletion of the available soil water stored before sowing, respectively.

Differences in SWD along soil layers were found among the six cultivars. New cultivars tended to have the ability to increase root growth in deeper soil layers under water stress conditions, resulting in a higher SWD. On average, for the three seasons, the new cultivar ‘Shimai 22’ had a higher proportion of root weight below the 60 cm soil layers than the old cultivar ‘Jimai 7’, and the soil water uptake below the 60 cm soil layer was 10.0-26.4% greater in the former than in the latter under MW and LW. The results showed that the new wheat cultivars significantly enhanced the capacity of soil water utilization in deep layers. Although the new cultivars tended to have smaller total root weights, the decrease in total root weight did not affect the effective use of soil water storage. The old cultivar tended to have a more extensive proliferation of roots in the topsoil, which did not increase the soil water use but increased the ineffective input in root growth.

**Figure 9:**
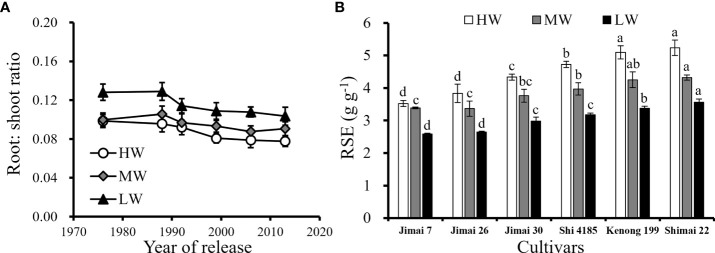
Changes in root: shoot ratio at harvesting with the released year of wheat cultivars **(A)** and average RSE for the six cultivars **(B)** under three water supply levels during the three growing seasons (Means ‘±’ standard deviation with different letters for the same irrigation treatment differ significantly at P < 0.05. HW, high-level water supply; MW, medium-level water supply; and LW, low-level water supply).

The difference in the root growth and biomass production among the cultivars resulted in the difference in the root: shoot (**Figure 9A**) and ultimately affected the root efficiency at the yield level **(** **Figure 9B** **)**. Although higher root weight density benefited soil water use, excessive root growth in the topsoil layers resulted in redundancy in roots without increasing soil water availability to cultivars released in earlier years. Excessive resource input in the root system was not conducive to the accumulation of photosynthetic products in grains, reducing the corresponding grain yield per unit root. That is, the root system efficiency (RSE) of ‘Shimai 22’ was approximately 27.6–48.6% higher than that of ‘Jimai 7’ under the three irrigation treatments, with a total root weight approximately 9.7-16.1% lower. The results indicated that an optimized root system structure with a decline in root biomass under HW and moderate proliferation of roots in deep soil layers under MW and LW treatments were beneficial for improving RSE.

Significant positive correlations (P < 0.01) were found between WP with HI and RSE (**Table 6**). Soil moisture under the HW treatment ensured a comparatively sufficient water supply during the growth period of winter wheat to delay leaf senescence and reduce the impact of Pn reduction during the reproductive stage on WP. A negative correlation between the root: shoot and WP indicated that excessive root growth in the topsoil layer, where roots are usually abundant, increased the carbon input in root growth, which was not conducive to the increase in soil water availability in deep soil layers and caused a waste of photosynthetic products. The results indicated that the WP of winter wheat was affected by both above- and below-ground characteristics.

**Table 6:** Correlation analysis of water productivity of winter wheat cultivars with the average Pn of flag leaves at grain filling (PnGF), harvest index (HI), proportion of soil water depletion in deep soil layers (SWD_d_), root: shoot (RS) and root system efficiency (RSE) under three irrigation treatments during the three growing seasons ※.

Water supply levels	PnGF	HI	SWDd	RS	RSE
High water supply	NS	+**	+*	-**	+**
Medium water supply	+**	+**	+*	-*	+**
Low water supply	+**	+**	+**	-**	+**

※: +: meaning positive correlation, -: meaning negative correlation. NS: not significant, *: significant at P < 0.05 level, **: significant at P < 0.01 level.

## 4 Discussion

### 4.1 Contribution of above-ground traits and dry matter allocation to high-yielding wheat cultivars

The results from this study indicated that yield and water productivity of new released wheat cultivars were significantly higher than that of earlier released cultivars under a range of water supply conditions (**Figures 1**–**3**). Many studies have indicated that the biomass accumulation after anthesis play an important role in achieving high grain production of winter wheat especially under water stress condition (Liu et al., 2016; Yang et al., 2021). It has been shown that each additional millimeter of water extracted by crops during reproductive stage of winter wheat can produce an additional grain yield of 55 kg ha^-1^ in the arid environment (Manschadi et al., 2006). Zhang et al. (2009) reported that 60-80% of seeds weight came from the dry matter accumulation of leaf photosynthesis during anthesis and grain filling stage of winter wheat. The comparison of the six wheat cultivars further showed that an earlier anthesis date and maintaining a relatively high Pn at grain filling stages under various irrigation treatments are important traits related to higher biomass accumulation and yield improvement by cultivar renewal (**Figures 5**, **6**). The results of this study agreed with previous reports that post anthesis dry matter production was significantly related to the improvement in yield, while the leaf senescence of the old cultivars sensitive to water stress conditions accelerated the degradation of stored dry matter, ultimately limiting dry matter accumulation and final yield (Abbasi et al., 2014; Sade et al., 2018; Fang et al., 2021).

Grain yield of winter wheat was both affected by the biomass production as well as the harvest index. Harvest index is generally increased with the shortening in plant height. The results from this study showed that the average value of plant height at maturity decreased from 65.9 cm to 62.3 cm for the six wheat cultivars released from the 1970s to 2010s (**Figure 4A**). Wheat cultivar stabilized its grain yield at the cost of shoot biomass *via* a significant decrease in plant height, suggesting a potential for partitioning of biomass to seeds characterized by HI is an important drought tolerance trait frequently found for cereals (Dreccer et al., 2009; Beche et al., 2014). Artificial selection for high-yielding cultivars indicated that shorter plants allocated fewer resources to grow in height and more to reproduction (Herder et al., 2010). Therefore, dwarfing alleles were integrated into high durum wheat by crossing semi-dwarfing bread wheat, achieving significant improvement in grain yield (Elazab et al., 2016).

The increased HI by cultivar renewing in this study was not only related to the reduction in plant height, but also to the increased efficiency in dry matter assimilation (**Figures 7A, B**) and allocation of carbon products to seeds (**Figure 4B**). The findings were consistent with others, such as Zhang et al. (2010); Zhang et al. (2013) and Fang et al. (2017) found that the stable and high yield of new winter wheat cultivars under various irrigation treatments was attributed to the increased efficiency in dry matter accumulation and allocation of carbon products to grains. The improvement in HI of new wheat cultivars was the result in the improved allocation of photosynthetic products to seeds, and the annual genetic gain with cultivar renewing has been proved consistently and positively associated with the grain weight and harvest index (Sun et al., 2014).

The increased WP at grain yield level for the new released cultivars as compared to the earlier released ones could be explained by the increase in biomass production and HI under similar water consumption, and in which HI played an important role in the increased WP. Water productivity at grain yield level is decided by the equation of biomass*HI/ET, the increased HI by the cultivar renewing significantly benefited the improvement in WP. Cultivar renewing not only increases the yield potential, but also WP. Higher WP means less water use for similar yield or increased yield without apparent increase in water consumption, which would be quite important for food security under increased water shortage situations around the world. New wheat cultivars could restrict luxury transpiration over the growing season through a more conservative water uptake strategy under water stress conditions, and tended to achieve higher efficiency in dry matter assimilation and water productivity (Richards et al., 2010).

### 4.2 Responses of root-related drought-adaptive characteristics among different winter wheat cultivars

Roots play an important role in the uptake of soil water and nutrients to meet the requirements of the above-ground parts. Maintaining a large root system would cost a lot of carbon products from the above-ground part. Therefore, an economic root system would be preferred. The results from this study indicated that cultivar renewing decreased the root: shoot ratio, but not the efficiency in utilization of the soil water (**Figure 9**). Continued breeding and cultivar testing in high input conditions removes constraints that would reveal root plasticity and selection of cultivars with ideal responses (Schneider and Lynch, 2020). The process of breeding shorter wheat cultivars may have produced indirect responses such as the reduction in root biomass and root: shoot ratio (Zhang et al., 2009; Bai et al., 2013).

The below-ground traits related to grain yield among wheat cultivars, including root distribution and root: shoot ratio, are affected by the genetic background and soil moisture (Fang et al., 2017; Nakhforoosh et al., 2021; Bacher et al., 2021). Soil water availability is the major limiting factor for yield improvement under deficit water supply (Wu et al., 2020). The root system architecture (spatial distribution of root length and biomass), as well as its anatomical and hydraulic properties, regulates plant water flow and maintains the whole plant water balance (Sivasakthi et al., 2017). The results from this study indicated that the limited-irrigation stimulated roots to grow into deeper soil layers and thus enhanced the uptake of soil-stored water from the subsoil layer (**Figure 8**), and there was higher density of root system and soil water utilization in deep soil layers for the new released wheat cultivars. Under deficit irrigation, water consumption during vegetative growth of winter wheat would results in insufficient available water for crops during the reproductive stage (Liang et al., 2021). Previous studies have shown that both yield and WP of winter wheat could be improved by increasing subsoil water consumption, which can increase available water for crops, especially during the reproductive growth stage (Lilley and Kirkegaard, 2011; Xu et al., 2016; Figueroa-Bustos et al., 2020).

Phenotypic plasticity of root: shoot ratio represents an adaptive mechanism for plants to water stress, that the root growth is favored over the shoot to limit evaporation and extract water residuals under drought (Correa et al., 2019; Carminati and Javaux, 2020). However, when the total amount of carbon assimilates is limited under water deficit, the accumulation of a larger proportion of carbohydrates in root tissues that maximize individual fitness in nature was one of the reasons that reduced the yield of old cultivars (Ma et al., 2008; Qin et al., 2019; Fradgley et al., 2020). In contrast, the root biomass in total dry matter (root + stem) was decreased among new cultivars with lower sensitivity to water stress conditions, and an optimized root system structure could efficiently uptake soil water under water stress conditions and allocate more biomass to grains characterized by higher RSE (**Figure 9B**). The results of this study were similar to those of Friedli et al. (2019) and Qi et al. (2019), who reported that the reduction in plant height of renewed cultivars significantly reduced the size of roots, thereby reducing the root: shoot ratio among wheat cultivars under various water supply conditions (**Figure 9A**). The gradual decrease in root biomass of wheat cultivars introduced over the last 50 years of breeding processes to increase grain yield by reducing nutrient allocations to root growth (Aziz et al., 2017). In general, cultivars with economical root structure under specific circumstances are conducive to the improvement of grain yield (Van Oosterom et al., 2016; Lynch, 2018; Boudiar et al., 2021).

### 4.3 Comprehensive regulation of below- and above-ground parts of dryland wheat cultivars to improve yield and water productivity

Results from this study indicated that new released cultivars out-performed the earlier released cultivars in both the below- and above-ground part of plants. For the above-ground part, the new released wheat cultivars produced more biomass and had higher efficiency in allocation of the dry matter to grains, resulting in a higher HI under various water supply conditions. For the below-ground part, the new cultivars reduced the input in root growth, but without compromising the efficiency in utilization of the soil water, which resulted in the reduction in root: shoot ratio and improved root efficiency. Plant species have evolved specialized strategies to regulate their above-ground biomass (AGB) and below-ground biomass (BGB), thereby affecting carbon (C) inputs and cycling (Sierra et al., 2017). Optimal partitioning theory (OPT), which assumes that there are trade-offs in biomass allocation between AGB and BGB, suggests that plants allocate biomass to acquire the limited resource and achieve the maximum yield with the renewal of cultivars (Ledo et al., 2018).

Previous studies indicated that the leaf photosynthetic rate was regulated by both hydraulic and non-hydraulic root-sourced signal (Lawlor and Cornic, 2002; Kong et al., 2013). There was positive correlation between leaf stomatal conductance and gas exchange rate with water uptake by root system (Fang et al., 2017). Deep, wide and multibranched roots are ideal target traits for soil water utilization in the breeding processes of drought-tolerant wheat cultivars, but there are trade-offs in corresponding grain yield (El Hassouni et al., 2018; Carminati and Javaux, 2020). Excessive growth of roots in topsoil accelerated the depletion of soil water storage in early growth stage of winter wheat, competition from adjacent roots promoted the production of abscisic acid and triggered reactive oxygen species generation, all of which would negatively affected grain production (Batool et al., 2019). During anthesis and grain filling stages, photosynthetic products are mainly transported to grains instead of roots (Woo et al., 2021). Maintain a large root system consuming a large amount of photosynthetic assimilates and negatively affecting crop production (Mehrabi et al., 2021). Newly released wheat cultivars with thicker roots in the deep soil layers and reducing roots in topsoil had a greater ability to penetrate deeper soil layers; thus, plants have access to more soil water storage under limited water supply (**Figure 8**), resulting in relatively higher Pn during the reproductive stage with more dry matter production for yield formation than cultivars that could not efficiently utilize stored water (Rebetzke et al., 2016; Figueroa-Bustos et al., 2020).

The results of this study were carried out under tube conditions, and the correlations between specific traits with grain yield and WP within tubes were not identical to those obtained from the field (Zhang et al., 2009; Fang et al., 2017; Lu et al., 2020). The reason for difference in results could be attributed to the proliferation of below- and above-ground parts of crops responding to adjacent plant structures in the field (Chen et al., 2021). Wheat cultivars that are similar to their wild ancestors have strong competitiveness, while artificial selection for high-yielding cultivars weakens the competitiveness of individual plants with smaller root sizes and the overlap between the canopies of nearby plants, indicating that the results obtained from wheat cultivar breeding processes under a control environment should be adopted for population selection in the field (Weiner et al., 2017; Zhu et al., 2022).

Another factor that might affect the experimental results in this study was that the growing conditions in the tubes was different from that in the field, due to the restrictions to the root growth by the tube wall. The soil physical properties of the repacked soil inside the tube might also affect the root growth. Valentine et al. (2012) reported that the elongation of roots in repacked soil was much less than that in the field due to differences in the continuous pore network among soil layers. Therefore, in the processes of winter wheat breeding, either high-yield traits of a single plant or the yield formation of plant groups should be considered synchronously under various environmental conditions.

## 5 Conclusions

Breeding processes of cultivars with stable yield under water stress conditions are of great importance to improve the yield and WP of winter wheat. The results from this study showed that the increased HI and reduced plant height among wheat cultivars released from the 1970s to 2010s were important factors for the improvement in grain production. New wheat cultivars could make effective use of deep soil water storage by increasing the proportion of deep roots and reducing the redundant root growth in the topsoil layer without increasing the input to root growth. Full utilization of soil water storage in deep soil layers among new cultivars maintained a relatively higher leaf photosynthesis rate and extended the reproductive stage through an earlier anthesis date, increased biomass production and allocation to seed weight and achieved higher yield than old cultivars. The root: shoot ratio decreased with the renewal of wheat cultivars, resulting in more photosynthetic products distributed to grains under limited resources supplemented by higher RSE and WP. The results of this study indicated that breeding high yield and high resource use efficiency winter wheat cultivars need to balance the growth of above- and below-ground parts of plants.

## Data availability statement

The raw data supporting the conclusions of this article will be made available by the authors, without undue reservation.

## Author contributions

LS conceived of and designed the study. HL analyzed the data and wrote the manuscript. LL, NL, and ZL performed the field measurements and samples collection. YL assisted with manuscript writing and editing. All authors approved the final version of the manuscript.

## Funding

This work was supported by National Key Research and Development Project of China (2017YFE0130500) and Hebei innovation group project (D2021503001).

## Conflict of interest

The authors declare that the research was conducted in the absence of any commercial or financial relationships that could be construed as a potential conflict of interest.

## Publisher’s note

All claims expressed in this article are solely those of the authors and do not necessarily represent those of their affiliated organizations, or those of the publisher, the editors and the reviewers. Any product that may be evaluated in this article, or claim that may be made by its manufacturer, is not guaranteed or endorsed by the publisher.
